# Sodium-Glucose Cotransporter 2 (SGLT2) Inhibitors: Benefits Versus Risk

**DOI:** 10.7759/cureus.33939

**Published:** 2023-01-18

**Authors:** Bains Jasleen, Gupta K Vishal, Malepati Sameera, Molla Fahad, O'Brien Brendan, Santander Deion, Sudhakar Pemminati

**Affiliations:** 1 Department of Biomedical Education, California Health Sciences University College of Osteopathic Medicine, Clovis, USA

**Keywords:** sglt-2, pulmonary, renal, cardiovascular, gliflozins

## Abstract

With the growing burden of metabolic disease, cardiovascular disease, and diabetes mellitus, there is an implication for new pharmacological intervention. Sodium-glucose cotransporter 2 inhibitors (SGLT2i) are a class of drugs that work on SGLT2 receptors in the kidneys to decrease glucose reabsorption. Lowering glucose levels mainly aids those with type 2 diabetes (T2DM), but they also have many other effects on the body. This article will investigate the impact of SGLT2i on six relevant organ systems; to establish current knowledge and potential benefits and risk for SGLTi in clinical practice. The medications that inhibit SGLT2 suffix with flozins are known to help decrease hypertension, acute cardiac failure, and bradycardia in the cardiovascular system. Flozins were found to aid with acute pulmonary edema, asthma, bronchitis, and chronic obstructive pulmonary disease (COPD) in the pulmonary system. SGLT2 is also found in the blood-brain barrier (BBB), and as such, SGLT2i can also affect the central nervous system (CNS). They reduced reactive oxygen species (ROS), BBB leakage, microglia burden, and acetylcholinesterase (AChE) levels. In the liver, this class of drugs can also assist with non-alcoholic fatty liver disease (NAFLD), hepatotoxicity, and weight loss. In the pancreas, SGLT2i has been shown to help with primarily diabetes and hyperglycemia. Finally, SGLT2i's are known to aid in decreasing nephrotoxicity and stopping the progression of the glomerular filtration rate (GFR) decrease. New studies have shown that the flozin drugs have been helpful for those who were receiving kidney transplants. Despite the positive effects, there are some concerns about SGLT2i and its notable adverse effects. Flozin drugs are known to cause urinary tract infections (UTIs), dehydration, orthostatic hypotension, postural dizziness, syncope, hypotension, hyperkalemia-induced cardiac arrest, and pancreatitis. This literature review will discuss, in detail, the benefits and risks that SGTL2i have on different organ systems and implicate the role they may play in clinical practice.

## Introduction and background

With the increasing incidence of fast-food restaurants, carbohydrate-dense foods, and food deserts, hyperglycemia is a global epidemic, affecting almost one billion individuals worldwide [1]. Persistent states of hyperglycemia chronically can lead to diabetes mellitus, the most prevalent chronic condition globally, affecting 10% of people and causing a public health burden financially [1]. Many classifications of pharmaceuticals have been formulated and released throughout the past century to help modulate blood glucose levels and help preserve these patients' vasculature and vital organs. In 2013, the first sodium-glucose cotransporter 2 SGLT2 inhibitor (SGLT2i), canagliflozin, was released after Food and Drug Administration (FDA) approval, followed by a cascade of similar "flozins" within the next several years [2]. The mechanism of SGLT2i is as follows: they modulate the sodium-glucose cotransporter on the nephron, inhibiting glucose reuptake, and allowing increased excretion of glucose in the urine, thus lowering the serum glucose levels [2]. Whether these drugs are taken individually or in conjunction with other glucose-modulating drugs (i.e., metformin, dipeptidyl peptidase 4 (DPP-4) inhibitors, glucagon-like polypeptide 1 (GLP-1) agonists, insulin), the effects of SGLT2i on glucose are heavily researched, showing significant benefits in maintaining optimal glucose homeostasis [3, 4]. With SGLT2i being available to patients for less than one decade, it is only recently that scientists have assessed some of its long-term effects on the human body. As studied for decades, diabetes is known to lead to harmful comorbidities and sequelae of complications. Therefore, it is essential to assess the benefits and risks of glucose-modulating drugs to ensure they do not exacerbate the effects of diabetes and are more protective of organ systems than destructive ones. This literature review examines the positive and negative impacts of SGLT2i on six crucial organs and systems in the body. These systems include the nervous, cardiovascular, pulmonary, pancreas, hepatic, and renal. We examined 35 scholarly articles from Google Scholar and PubMed databases after 2017 that were relevant to SGLT2i and any individual or combination of organs. We included randomized controlled trials, systematic reviews, meta-analyses, and traditional reviews.

## Review

Benefits and risks on the central nervous system

Sodium-glucose cotransporters 2 (SGLT2) are widely found in various brain regions, such as the hippocampus, cerebellum, and blood-brain barrier (BBB). These biologically essential transporters in the brain make them a potential target in treating many central nervous system disorders [5]. As such, SGLT2i are being extensively studied for potency in preventing or protecting against certain neurological diseases. Three main mechanisms that link SGLT2 inhibitors with cognitive function are that SGLT2i reduces reactive oxygen species (ROS), lessens leakage of BBB, and decreases microglia burden and acetylcholinesterase levels [6]. Specific SGLT2i's are further discussed in this literature to discover their properties and how they impact cognitive health. 

Previous research studies have discovered three crucial inhibitors from the flozin family: empagliflozin, eapagliflozin, and canagliflozin. Empagliflozin has the highest sensitivity for SGLT2 at 2500-fold [7]. Dapagliflozin and canagliflozin have lower sensitivity at 1200 and 250-fold, respectively [7]. The sensitivity of empagliflozin and dapagliflozin has proven to be high and is further discussed in this literature study. 

Recent research suggests that SGLT2 inhibitors influence Alzheimer's disease pathology and brain injury caused due to ischemic-inducing factors [5]. Empagliflozin was reported to reduce senile plaque density and soluble and insoluble amyloid beta levels in APP/PS1xd/db model mice [5]. SGLT2 inhibitors were also noted to enhance angiogenesis and neurogenesis and attenuate ischemia-related central nervous system (CNS) damage. Empagliflozin increased the levels of H1F-1 alpha and VEGF-A and decreased the levels of caspase-3. Caspase-3 levels decreased by 85% with empagliflozin treatment in mice induced with ischemia/reperfusion injury [8]. It is proposed that empagliflozin exerts its neuroprotective effect by gaining entry into the disrupted BBB. SGLT inhibition is linked with the downregulation of atherosclerotic disease, which is responsible for causing cerebral ischemic events. Reports suggested that empagliflozin limits atherosclerotic plaques in the aortic arch and valve in ApoE-/- mice model. Empagliflozin also downregulated the concentration of tumor necrosis factor (TNF)-alpha, interleukin 6 (IL-6), and monocyte chemoattractant protein-1 (MCP-1). Empagliflozin decreased infarct volume by attenuating cerebral oxidative stress, inflammation, and apoptotic markers. Empagliflozin reduced oxidative stress by decreasing malondialdehyde (MDA) and increasing catalase and glutathione (GSH) concentration [5]. GSH and catalase levels increased by 0.36 and 2.3-folds primarily due to empagliflozin treatment in mice subjected to a cerebral injury [8]. Lastly, empagliflozin also exhibits neuroprotective effects against hyperglycemia and prevents any change in the microvascular structure of brain cells [7].

**Figure 1:**
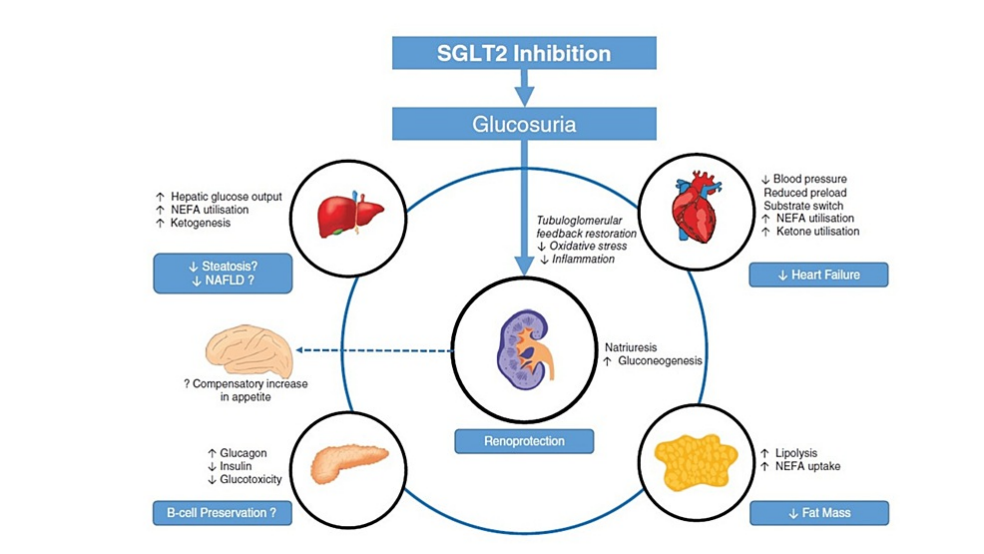
The effects of SGLT2 inhibitors on various systems of the body The figure is reproduced with permission [37] SGLT2 - sodium-glucose cotransporter 2, NEFA - non-esterified fatty acid, NAFLD - non-alcoholic fatty liver disease

Dapagliflozin effectively reduced seizure activity in rats induced with pentylenetetrazol (PTZ) [9]. Without treatment, the PTZ-treated arm had a spike wave percentage (SWP) of 75.3%, and SWP decreased drastically to 20.4% after treatment with 75 mg/kg of dapagliflozin. A further reduction of 6.1% in SWP was measured when the dosage of dapagliflozin increased to 150 mg/kg. The addition of dapagliflozin significantly lowered the Racines scales score (RSS) by 2.33 +/- 0.5 in rats treated with 75 mg/kg dosage and by 2.1 +/- 0.5 in the 150 mg/kg dosage group. Alone with PTZ, time to first myoclonic jerk (TMFJ) reached a mean of 68.3 sec; with dapagliflozin 75 mg/kg dosage, the TFMJ advanced to a standard of 196.7 sec, and with 150 mg/kg of dapagliflozin, TFMJ reached its highest at 268.3 sec [9]. In addition to protecting against epilepsy, dapagliflozin also prevented neuronal injury and motor dysfunction in rats induced with Parkinson's disease [6]. Dapagliflozin was also noted to minimize stroke risk factors by reducing weight and visceral fat in rats, leading to improved insulin sensitivity and decreased calorie intake, which was attributed to increased glycosuria. Dapagliflozin decreased malondialdehyde (MDA) levels in obese rats, indicating reduced oxidative stress. Dapagliflozin also showed antiapoptotic activity by reducing Bax and increasing Bcl2. When combined with liraglutide, dapagliflozin also increased neurogenesis and synaptic density in diabetic mice [7]. Overall, these findings suggest the benefits of using empagliflozin and dapagliflozin for not only hyperglycemia but many neurological conditions. In addition, it should be noted that zero to minimal risks were reported of SGLT inhibitors on the brain, indicative of further research required in this fairly novel research field. The findings are summarized in Table 1 and Figure 1.

**Table 1:** Benefits and risks on the central nervous system SGLT2 - sodium-glucose cotransporter 2

Author	Country	Study population	Benefits	Risks
Tharmaraja et al., 2022 [6]	Meta-analysis	160 studies	SGLT2 inhibitors enhanced memory deficits which were primarily attributed to diabetes, reduced epilepsy and severity of seizures, lowered the risk of dementia, and a prominent reduction was observed in neuronal cell death (these effects were observed in laboratory mice models).	Due to the extreme novelty in this research area and the paucity of human studies, not many risks are reported.
Amin et al., 2020 [8]	Egypt	40 laboratory rat models	After administration of SGLT2 inhibitors, improvement in terms of behavioral and neurological functions were observed in rat models induced with cerebral ischemia/reperfusion injury.	N/A
Erdogan et al., 2018 [9]	Turkey	48 laboratory rat models	Dapagliflozin showed its neuroprotective effects by decreasing Spike wave percentage (SWP), reducing mortality rates, lowering the Racine Scales Scores (RSS), and drastically increased the time to first myoclonic jerk (TFMJ) in mice with induced seizures.	Further clinical research recommended to determine clinical risks

Benefits and risks on the cardiovascular and pulmonary systems

Sodium-glucose cotransporter-2 inhibitors (SGLT2i) have been used to prevent the development and progression of various cardiovascular and respiratory diseases. Several placebo-controlled trials suggest that adverse cardiovascular events were reduced after treatment in patients with type 2 diabetes [10-13]. This drug class is known to impact the myocardium and provide a protective effect by reducing the secretion rate of proinflammatory chemokines [13]. This outcome is primarily due to SGLT2i's impact on reducing body weight, specifically body fat. A recent meta-analysis of eleven cardiovascular outcomes trials involving 77,541 patients assessed patients' death and hospitalization rates using dapagliflozin and empagliflozin. The study reported decreased death and hospitalization rates independent of type 2 diabetes status [12]. This claim demonstrates their utility as a medication for improving the prognosis for cardiovascular complications [12]. Some additional cardiovascular benefits obtained from SGLT2i use include blood pressure lowering effects of systolic and diastolic blood pressure [14, 15]. A meta-analysis of nine large trials demonstrated that SGLT2i are associated with decreased incidence of nine types of cardiovascular disease and eleven types of respiratory disease [16]. The cardiovascular diseases with decreased incidences included conditions such as atrial fibrillation, bradycardia, hypertension, hypertensive emergency, acute cardiac failure, and varicose veins [16]. The respiratory diseases associated with decreased incidence with SGLT inhibitor use included acute pulmonary edema, asthma, bronchitis, COPD, non-small cell lung cancer, pneumonia, pleural effusion, pulmonary edema and masses, respiratory tract infections, and sleep apnea syndrome [16].

The adverse effects of SGLT2 inhibitor use are symptoms accompanying the reported osmotic diuresis, such as volume depletion, dehydration, orthostatic hypotension, postural dizziness, syncope, and hypotension [11]. Although severe, these side effects are reported as infrequent and occur with a higher incidence in elderly patients, those on high drug dosages, those who use loop diuretics, and individuals with kidney dysfunction [11]. The secondary effects of this drug can lead to excessive weight loss and lowering of blood pressure which may be undesirable and must be considered [17]. One article reported a mild to moderate increase in urinary tract infections with SGLT2 use, with *Candida *infections being the most common adverse effect of SGLT inhibition [11]. The EMPAG-Outcome Trial in 2015 has also demonstrated that compared to placebo, the use of empagliflozin leads to similar rates of adverse events, including acute renal failure, diabetic ketoacidosis, thromboembolic events, bone fracture, and volume depletion [18]. This trial also showed no significant difference in rates of myocardial infarction (MI) and stroke when comparing the placebo and empagliflozin groups. However, the empagliflozin group had a lower risk of death from cardiovascular causes, death from any reason, and hospitalization for heart failure patients [13, 18].

SGLT2 inhibitors can also significantly reduce the occurrence of acute pulmonary edema, asthma, and sleep apnea syndrome [19]. The reduced risks of respiratory disorders are associated with the glucose-lowering effects of SGLT2is; however, the mechanism for the reduction needs to be further researched [19]. Compared with the placebo, SGLT2 inhibitors have also been known to produce rapid reductions in pulmonary artery pressures [20]. This represents one of the most important measures of heart failure and is highly predictive of clinical events, including hospitalizations and death [20]. The direct beneficial effect of the SGLT2 inhibitors is seen in maintaining pulmonary artery pressures in the optimal range and has been shown to reduce the risk of such adverse events [20].

Another reported effect of SGLT2 inhibitors includes an increase in hematocrit level, which the researchers hypothesized to be secondary to plasma volume contraction and diuresis [11]. Other studies have also reported elevations in high-density lipoprotein (HDL) and low-density lipoprotein (LDL) cholesterol (C) and reductions in triglycerides associated with SGLT2 inhibitor use [11, 15, 18]. The peaks in HDL and LDL were similar, resulting in no change in the HDL-C to LDL-C ratio [15]. These claims have raised concerns regarding the potential cardiovascular consequence of using SGLT2 inhibitors. Researchers have stated that more attention is needed to determine whether these small increases in lipid levels are clinically relevant or may offset any potential cardiovascular benefits of SGLT2 inhibition [15]. Other reported effects of use include reductions in uric acid levels [11, 15, 18]. The impacts of this drug class have been confirmed by several relevant trials and have the potential to be associated with the reduced reduction of cardiovascular and respiratory diseases. The findings are summarized in Table 2 and Figure 1.

**Table 2:** Benefits and risks on the cardiovascular and pulmonary systems SGLT2 - sodium-glucose cotransporter 2, PA - pulmonary artery

Author	Country	Study population	Benefits	Risks
Zou et al., 2019 [13]	China	42 trials with a total of 61,076 patients	SGLT2 inhibitor treatment was associated with a reduction in the incidence of, myocardial infarction, cardiovascular mortality, and all-cause mortality.	The risk of ischemic stroke was not reduced after SGLT2 inhibitor treatment in patients with type 2 diabetes.
Ridderstråle et al., 2014 [14]	Denmark	Double-blind phase 3 trial, 1549 patients (aged ≥18 years) with type 2 diabetes and HbA1c concentrations of 7-10%.	SGLT2 inhibitors had the added benefit of lowering body weight and blood pressure, preserving the GFR, lower hypoglycemic adverse events when compared to glimepiride.	Small increases from baseline in hematocrit, total cholesterol, low-density lipoprotein cholesterol, and high-density lipoprotein cholesterol levels were noted with empagliflozin compared with glimepiride.
Yin et al., 2021 [16]	China	33,124 participants taking SGLT2is and 26,568 participants taking placebo	SGLT2i's were observed with reduced risks of infectious respiratory disorders, which might be associated with the glucose-lowering efficacy of the medication.	SGLT2is were not significantly associated with the reduction of the other 115 cardiovascular and respiratory diseases.
Nassif et al., 2021 [20]	United States of America	93 patients with heart failure	Empagliflozin significantly reduced PA diastolic pressure, with effects that began at week 1 and amplified over time with empagliflozin versus placebo. Results were consistent for PA systolic, and PA mean pressures.	Empagliflozin can significantly increase fractional sodium excretion, which persists for at least several weeks, and reduce blood volume and plasma volume.

Benefits and risks on the pancreas

Although the primary mechanisms of SGLT2 inhibitors occur in the nephron, their involvement with glucose regulation allows us to consider the implications on the pancreas. After reviewing gene expressions for SLC5A2 (which encodes for SGLT2), alongside changes in both insulin and glucagon through their correlated alpha and beta cells, these results provided insight into how glucose regulation occurs after these drugs are administered. 

The first impact on the pancreas involves glucagon since these drugs lead to glycosuria, which can lead to hypoglycemia, resulting in a cascade requiring alpha cells to secrete glucagon for homeostasis [21]. Additionally, SGLT2 proteins themselves are present in the pancreatic alpha cells. These SGLT2 transporters colocalize in the alpha cells with glucagon and not insulin or somatostatin in beta cells, displaying a direct relationship between glucagon and SGLT2 proteins when unregulated glucose levels are present [22]. This direct association allows for the development of pharmaceuticals that lead to fewer hypoglycemic events for individuals with both type 1 and type 2 diabetes who are potentially on multiple medications [23]. There is an increase in glucagon after administering SGLT2 inhibitors since decreasing glucose uptake leads to less glycolysis and more glycogenolysis and gluconeogenesis, reducing the amount of adenosine triphosphate (ATP) formed. The lack of ATP ultimately leads to repolarization and activation of calcium, sodium, and K-ATP channels to secrete glucagon, leading to hyperkalemia, which can have cardiac manifestations [23]. 

SLC5A2 expression and hepatocyte nuclear factor 4-A (HNF4A), which regulates the expression of SLC5A2, decrease significantly in episodes of hyperglycemia because alpha cells become idle and do not require glucagon secretion [24]. Of the many drugs, the primary SGLT2i that shows meaningful interactions with glucagon is dapagliflozin. In many literature sources, dapagliflozin has been shown to assist in glucagon secretion and liver metabolism, specifically gluconeogenesis, shortly after administration, preventing any hypoglycemic episodes that can be harmful or even lethal for some patients [21, 22]. Dapagliflozin also upregulates SGLT1 transporters, which help more in the uptake of dietary glucose versus the nephrotic reuptake, affecting the small intestine, too, leading to more optimal glucose homeostasis. 

After discussing the implications of the direct link between SGLT2 inhibitors and glucagon release, it is essential to understand the physiology behind insulin sensitivity and resistance, which leads to diabetic patients requiring many pharmaceuticals, including SGLT2 inhibitors. Although glucagon increases post-SGLT2-inhibitor administration to maintain serum glucose levels, the effects on insulin sensitivity appear counterintuitive but are also impacted by the effects of these drugs, especially empagliflozin and luseogliflozin. In numerous studies, researchers discovered that those administered SGLT2 inhibitors in the preliminary stages of diabetes or for long-term durations have physiologic and histological changes occurring in beta cells to alter insulin gene expression and sensitivity [24-26]. Through immunohistochemistry, substantial changes included an increase in beta cell mass and proliferation, alongside a decrease in beta cell apoptosis, demonstrating the beta cells' ability to adapt and alter their insulin resistance [24, 25]. These changes in beta cell mass result from increased gene expressions of insulin 1 and 2 and glucose transporter 2 (GLUT2) of many, which allow for enhanced insulin sensitivity and release in postprandial settings [24]. While SGLT2 inhibitors work adequately on their own, empagliflozin is more efficient with its mechanism when co-administered with a Dipeptidyl peptidase 4 (DPP-4) inhibitor, like linagliptin, leading to further improvements in glycemic control in an insulin-resistant state [25]. Another confounding result of the empagliflozin administration is the reduction of free-radical formation since less insulin is needed to be secreted due to the increase in sensitivity, leading to less generation of NADPH, phosphatidylinositol 3'-kinase-dependent free radical production [26]. 

After looking at the impact of SGLT2i on the pancreas, it is sure to say that both alpha and beta cells are stimulated through different mechanisms. Whereas glucagon is secreted due to the immediate hypoglycemic state due to the excretion of glucose through urine, this homeostatic window of glycemic control enables the insulin receptors to be sensitized. These two crucial hormones work in synchrony and lead to better glucose balance, lowering the risk of hyaline atherosclerosis and the significant long-term effects of diabetes on several vital organs. As for the risks of SGLT2i on the pancreas, there is literature stating that cases of pancreatitis were sporadically found in <1% of patients who were taking these medications, specifically dapagliflozin, but with diabetes and hyperglycemia already being a significant risk factor to pancreatitis, these effects can be due to confounding variables [21, 22]. The findings are summarized in Table 3 and Figure 1.

**Table 3:** Benefits and risks on the pancreatic islets SGLT2 - sodium-glucose cotransporter 2

Author	Country	Study population	Benefits	Risks
Bonner et al., 2015 [22]	France	3 groups of 10-14 islets (human islets, mouse islets, and human islets transplanted into immunodeficient mice.)	Those with type 2 diabetes have a lower blood glucose post-SGLT2i administration due to the increased glycosuria, compared to the placebo, leading to a more optimal homeostatic window for serum glucose levels.	In those with hyperglucagonemia, alpha-cell genes are upregulated after chronic SGLT2i administration which results in brief episodes of hypoglycemia. This leads to higher levels of glucagon, which can activate gluconeogenesis, contributing to increased serum glucose levels, even during fasting.
Saponaro et al., 2020 [23]	France	207 human islet preparations	SGLT2i decreases glycemia, increases the sensitivity of beta-cells, and reduces the risk of diabetes-related heart failure and other cardiovascular episodes.	Due to the relationship between glucagon and ketone bodies, critically ill patients can present with a significantly higher risk of developing diabetic ketoacidosis.
Cheng et al., 2016 [26]	China	24 male C57BL/6J mice (10 weeks of age); split into 2 groups of 12	SGLT2i improves insulin sensitivity, reverses glucotoxicity, regulates glucose levels, and reduces the risk of weight gain. There is also a reduction of oxidative stress within the beta-cells.	Risk of hypoglycemia post SGLT2i administration.

Benefits and risks on the hepatic system

The effect of SGLT2i on the hepatic system has been under investigation for its potential metabolic benefits. SGLT2i's are a drug class with the goal of improving glycemic control in patients with type 1 and type 2 diabetes mellitus (DM). Randomized controlled clinical trials show that these drugs can reduce Hemoglobin A1C (HbA1c) levels in diabetic patients and reduce the insulin dosage needed to sustain lower blood glucose levels [27]. Specifically, these drugs have shown low levels of adverse events associated with other glycemic control medications, namely hypoglycemia and diabetic ketoacidosis (DKA) [27]. SGLT2i works by preventing glucose reabsorption in the kidney's proximal convoluted tubule, allowing for excretion in the urine. This mechanism is independent of insulin and shows promise for improving BMI and cardiovascular outcomes [27]. 

SGLT2i has also shown potential for improving liver biomarkers, liver injury recovery, and hepatic fibrosis and steatosis [28, 29]. Non-alcoholic fatty liver disease (NAFLD) is the presence of fat accumulation in the liver from causes other than alcohol, drugs, or hypothyroidism. A recent systematic review found that multiple different SGLT2i compounds were associated with improvements in NAFLD in patients with type 2 diabetes mellitus (T2DM). It remains to be seen if the improvement mechanism is metabolically mediated or is a direct effect of the SGLT2i compounds [28]. 

T2DM has an increased risk of NAFLD following changes due to insulin resistance and changes in metabolic markers. In NAFLD, the constant high glucose levels from T2DM increase lipogenesis in hepatic tissue. The proinflammatory adipokines released in this disease state alter liver normal liver function [30]. NAFLD is associated with cardiovascular disease risk and hepatocellular carcinoma and currently has limited therapy options outside of glucose-lowering agents [30]. A recent trial showed that the combination of exenatide and dapagliflozin improved liver enzymes and fatty liver biomarkers in T2DM patients compared to exenatide alone or placebo [30]. Additionally, combination treatment resulted in more significant weight loss than using either agent independently or a placebo in T2DM patients with NAFLD. It has been shown that weight loss is correlated with an improved NAFLD activity score [31]. 

Another recent randomized controlled trial showed that dapagliflozin, an SGLT2i, decreased liver fat and volume in T2DM patients in just eight weeks of treatment compared to a placebo. The decrease in fibroblast growth factor-21 (FGF21) may be associated with the reduction in liver fat in these patients. It has also been recognized that a decrease in FGF21 improves mitochondrial function [32]. This intervention also significantly decreased visceral adipose tissue (AT) and inflammatory biomarker Interleukin-6 (IL-6). The decrease in visceral AT is corollary with the loss of liver fat and improved NALFD. The significance of decreased IL-6 is that it is associated with myocardial infarctions when it is in high concentrations in the blood. Additionally, there is a significant reduction in HbA1c and BMI, despite no changes in lean body mass [32]. These findings suggest that SGLT2i is a promising compound not only for the treatment of hyperglycemia but also for metabolic disorders. The findings are summarized in Table 4 and Figure 1.

**Table 4:** Benefits and risks on the hepatic system T1DM - type 1 diabeses mellitus, T2DM - type 2 diabetes mellitus, SGLT2 - sodium-glucose cotransporter 2

Author	Country	Study population	Benefits	Risks
Dandona et al., 2017 [27]	United States	833 patients with inadequately controlled T1DM on insulin	Dapagliflozin improved HbA1c and decreased dependence on insulin.	Risk of nasopharyngitis, urinary tract infection, respiratory tract infection, hypogylcemia.
Kinoshita et al., 2018 [29]	Japan	156 patients with T2DM	Patients on SGLT2i had improved glycemic control and liver injury recovery.	Risk of sarcopenia in older adults with diabetes is a concern due to decreased body mass.
Gastadelli et al., 2019 [30]	Italy	685 patients with T2DM and liver disease	Exenatide with dapagliflozin improved liver enzymes of hepatic steatosis and fibrosis in patients with T2DM.	The histological features of the liver after SGLT2i treatment in this study have yet to be examined.
Latva- Rasku et al., 2019 [32]	Finland	32 patients with 3 months of stable treatment of metformin or dipeptidase inhibitors (HbA1c 6.5-10.5%)	After dapagliflozin treatment there was a decrease in liver volume, improved glycemic control compared to placebo.	There was no change in insulin-stimulated glucose uptake in any tissues.

Benefits and risks on the renal system

As with other systems, SGLT2i has both positive results for kidney diseases and notable side effects. SGLT2i affects the renin-angiotensin-aldosterone system (RAAS) pathway in the kidneys by decreasing the renin pathway through reduced sodium reabsorption. Downregulating this pathway allows for renoprotection by inhibiting hyperfiltration injuries [33]. SGLT2 inhibitors excrete glucose by decreasing reabsorption through the SGLT2 channels in the proximal convoluted tubules [34]. In patients with diabetic kidney disease (DKD), the renin-angiotensin-aldosterone (RAA) axis is upregulated, which results in many progressive kidney diseases. DKD patients have upregulated angiotensin 2 (ANG2) and, therefore, the SGTL1/2 receptors [4]. The excretion of glucose can regulate blood sugar in patients who have diabetes [35]. SGLT2 inhibitors decrease glucose reabsorption in the kidneys and increase muscle-specific insulin sensitivity [34]. These effects help lose calories and decrease weight by excreting glucose. 

While diabetes is usually associated with insulin resistance, chronic kidney disease (CKD) has also led to insulin resistance due to increased proinflammatory cytokines, metabolic acidosis, aldosterone, urea, and urea toxin [34]. As such, physicians and researchers can expect that SGLT2 inhibitors will positively impact those with CKD. Dapagliflozin, an SGLT2 inhibitor, has positively affected patients with CKD by decreasing albuminuria and the progression of CKD [34]. Meanwhile, the EMPA-REG Outcome trial showed that empagliflozin also decreased macroalbuminuria by up to 55% compared to the placebo [35]. Another study, the CREDENCE trial, showed that canagliflozin reduced the risk for end-stage kidney disease and creatinine doubling by at least 32% [35]. 

Furthermore, SGLT2 inhibitors also lower intraglomerular pressure and glomerular hyperfiltration, which helps maintain the estimated-glomerular filtration rate (eGFR) in patients with kidney disease [10]. Studies show that canagliflozin decreases glomerular sclerosis, mesangial expansion, tubule-interstitial fibrosis, and intratubular cast formation caused by ANG2 infusions [4]. Traditionally, losartan is used to block the ANG2 receptor, but when paired with empagliflozin, losartan's effects were shown to increase [4]. A novel use of SGLT2i is its aid in patients who received kidney transplants. While the data is limited, SGLT2i seem to aid with glycemic control, body weight, and uric acid levels in kidney transplant recipients (KTR) [36]. 

Side effects should also be considered when prescribing SGLT2is. A study found that DM2 patients who took SGTL2 inhibitors may have possible acute kidney injury (AKI), especially when taken with non-steroidal anti-inflammatory drugs (NSAIDs), anti-Ras, or diuretics [34]. Dapagliflozin has been cited for advancing renal dysfunction [33]. Finally, studies show that kidney transplant recipients (KTRs) are more likely to have UTIs because of physiological changes in the GU system and increased euglycemic ketoacidosis due to SGLT2 inhibition [36]. While SGLT2i may have some negative side effects, this class of drugs shows promising results for many patients with CKD, lowering GFR, and albuminuria. The therapeutic uses of SGLT2i are summarized in Table 5 [10]. The findings are summarized in Table 6 and Figure 1.

**Table 5:** Therapeutic uses of SGLT2 inhibitors Adapted from reference [10]; "+"  - indicated, "-" - not indicated, SGLT2 - sodium-glucose cotransporter 2

Indications	Canagliflozin	Dapagliflozin	Empagliflozin
Type 2 diabetes mellitus	+	+	+
Reduction of cardiovascular mortality	+	-	+
Heart failure	-	+	+
Diabetic nephropathy	+	-	-
Chronic kidney disease	-	+	-

**Table 6:** Benefits and risks on the renal system CV - cardiovascular, T2DM - type 2 diabetes mellitus, eGFR - estimated glomerular filtration rate, SGLT2 - sodium-glucose cotransporter 2, CKD - chronic kidney disease, AKI - acute kidney injury

Author	Country	Study population	Benefits	Risks
Garofalo C et al., 2019 [29]	Italy	4 studies EMPA-REG, CREDENCE, DECLARE, and CANVAS with a total of 34000 patients, some with CV risk and T2DM	The study showed that there was a decrease in albuminuria and maintained eGFR.	Studies showed adverse effects of falls, UTIs, and acute kidney injury. Canagliflozin was a higher possibility of amputations.
Ujjawa Al et al., 2022 [36]	Review	7363	Meta-analysis showed that SGLT2i helped patients with CKD.	SGLT2i recipients may have an increased risk for euglycemic ketoacidosis, AKI, and bone fractures.

SGLT2i effect on obesity

Due to a direct positive correlation between type 2 diabetes and adiposity, many patients taking SGLT2is simultaneously try to lose weight to help with their prognosis. Since the SGLT2i mechanism inhibits glucose absorption in the renal tubules and excretes glucose in the urine, this ultimately results in the loss of calories, creating a negative energy balance [38, 39]. Some articles stated that through trials, patients on empagliflozin would have an energy loss of around 200 kcal/day due to this being a combination of the mechanism and increased B-oxidation, resulting in patients losing ~3 kg (6.6 pounds) in roughly a year and increased insulin sensitivity [38, 39]. Other studies claim that SGLT2i's, in conjunction with GLP-1 agonists, can lead to increased weight loss through genetic weight loss markers which results in a loss of up to 5 kg (11 pounds) [40]. Overall, SGLT2i's can aid in weight loss, leading to many benefits, including lower blood pressure, increased hematocrit, decreased hepatic fat, and more optimal glucose homeostasis [17, 36, 38-40]. 

## Conclusions

Through this review, we aimed to assess the benefits and complications of SGLT2i on the cardiovascular, pulmonary, nervous, hepatic, and renal systems, alongside the pancreas, in diabetic patients. There were significantly more protective factors than risk factors, demonstrating the high efficacy and usage of this class of drugs deemed beneficial to hyperglycemic individuals. Collectively, SGLT2i led to an overall decrease in mortality and a wide array of physiological processes that would add to these patients' longevity. These benefits include weight loss, liver enzymes and biomarkers improvements, optimal glucose homeostasis due to insulin and glucagon in the pancreas, less incidence of cardiopulmonary-related and neurological diseases, cardioprotective effects, and reductions in blood pressure. Although this paper's sections focused explicitly on each organ/system's many benefits, some adverse risks in a small proportion of individuals on SGLT2i include volume depletion and hypotension, pancreatitis, and episodes of temporary hypoglycemia. While these risks are potentially harmful to the patient, they occur in less than 1% of cases and can be due to confounding variables and preexisting patient health. In conclusion, SGLT2i act primarily on the nephron's proximal convoluted tubule (PCT). Still, there is also a tiny percentage of SGLTs in the kidney, brain, liver, thyroid, muscle, and heart, thus needing researchers to assess its holistic impact on individuals collectively. Through this literature review, we concluded that SGLT2s are beneficial in most patients, especially those with renal dysfunction, and we are optimistic about the future evolution of this drug class.
